# Challenges and opportunities of human resource management activities for neglected tropical diseases in Liberia

**DOI:** 10.1016/j.ssmhs.2025.100092

**Published:** 2025-12

**Authors:** Wede Seekey, Rosalind McCollum, Laura Dean, Hannah Berrian, Jerry Kollie, John S. Smith, Zeela Zaizay, Karsor Kollie, Emerson Rogers, Maneesh Phillip, Colleen Parker, Sally Theobald, Joanna Raven

**Affiliations:** a University of Liberia, Pacific Institute for Research and Evaluation, Monrovia, Liberia; b Liverpool School of Tropical Medicine, Liverpool, United Kingdom; c Actions Changing Lives, Monrovia, Liberia; d Ministry of Health, Monrovia, Liberia; e Effect Hope, Toronto, Canada

**Keywords:** Neglected tropical diseases, Human resource management, Human resources for health, Performance, Liberia, Qualitative research

## Abstract

People affected by skin neglected tropical diseases (NTDs) are best cared for by a motivated, well-directed, competent and well-resourced health workforce. There is limited evidence about performance management for health workforce relating to NTD tasks. We explored human resource management relating to skin NTDs, with a focus on performance management. We carried out qualitative and participatory research with health workers across health systems levels in Liberia to explore experiences of caring for people with skin NTDs and views on optimal human resource management (HRM) practices. We conducted key informant interviews with national health systems policymakers (16) and county health workers (32); in-depth interviews with health workers (36); focus group discussions with health workers (4); and photovoice with 15 community health assistants and community health promoters, purposively selected for maximum variation. All interviews and FGDs were transcribed and analysed using thematic framework approach. We found health workers often have strong intrinsic motivation to care for people affected by skin NTDs. However, this is undermined by weak HRM structures particularly in geographic areas where integrated services for NTDs requiring case management have not yet rolled out. The main challenges described include: limited awareness of NTD-related roles, and mental health support provision role, particularly at facility level, gaps in knowledge and skills (how to identify, diagnose and manage skin NTDs), irregular supervision and limited resources to deliver care. Our findings have informed collaborative development of a bundle of HRM approaches to strengthen performance of health workers caring for patients with skin NTDs, including participatory training informed by adult learning-based approaches, supportive supervision, provision of job tasks, NTD manual and related tools, essential resource provision for community health assistants and promoters (CHAs and CHPs) and non-cash awards.

## Highlights

• The health workforce is critical to ensuring person-centred care for persons affected by skin NTDs.
• Vrooms expectancy theory provides a useful framework for evaluating human resource management practices.
• Health workers’ intrinsic motivation to care for their patients is undermined by weak human resource management structures.

## Introduction

Neglected tropical diseases (NTDs) are an important global health challenge. They are preventable, and many are treatable with early detection and management. However, NTDs continue to affect around 1 billion people globally, primarily those who are most disadvantaged and marginalised, living in the poorest countries and communities (Kuper, 2019, Sun and Amon, 2018). Skin NTDs such as leprosy, Buruli Ulcer, yaws, lymphatic filariasis manifestations (hydrocele and lymphoedema)) and onchocerciasis are a recognized sub-group of NTDs. NTDs are frequently reported, diagnosed and treated late, by which time they have often led to chronic conditions, mental distress and disability (Dean et al., 2022, Kuper, 2019). There is increasing understanding of the psychosocial consequences of NTDs, with rates of common mental health conditions amongst persons affected by skin NTDs significantly higher than the general population (Dean et al., 2022). In order to provide person-centred which addresses both the physical and mental health needs of persons affected, health workers must be equipped with the knowledge, tools and skills needed to be able to diagnose, manage and refer persons affected for common mental health conditions. There are multiple and complex barriers to care-seeking from both a demand and supply side perspective (McCollum et al., 2022).

The health workforce is a critical component of the health system that underpins efforts to control and eliminate NTDs as well as care for people with NTDs. The quality of NTD service delivery relates closely to the work carried out by health workers, both skilled and voluntary. NTD elimination and control programmes are often highly reliant on the provision of services by voluntary Community Health Workers (CHWs). Providing an effective way to reach endemic communities with a range of interventions, NTD volunteers can have both positive or negative influence on the outcome of programmes (Krentel et al., 2017). These NTD volunteers do not work alone, but operate alongside a range of formally trained health workers as part of the broader health system. Additionally, many persons affected often first seek care from informal providers, including faith and traditional healers (McCollum et al., 2022), yet there is often distrust between formal and informal providers. Health workers at all levels need support to ensure that they fulfil their vital roles and contribute effectively to NTD programmes and health system strengthening (Scott et al., 2018, World Health Organization [WHO], 2018).

Liberia is one of many African countries where skin NTDs are endemic. Across all counties in Liberia, the burden of skin NTDs, namely Buruli ulcer, yaws, onchocerciasis, leprosy and lymphatic filariasis (resulting hydrocele and lymphoedema) is high among the general population (Chandler and Fuller, 2018, World health Organisation, 2019). Liberia became one of the first countries in the world to develop, adopt and begin implementation of a national integrated approach to managing skin NTDs in 2016 through their integrated case management NTDs plan. This integrated approach combines activities for multiple NTDs within a single approach for their detection, diagnosis and management. Reducing the burden of severe stigmatising skin diseases in Liberia (REDRESS) is a Ministry of Health (MOH), Liberia, led research programme aiming to strengthen integrated case management and improve the care of people affected by skin NTDs in Liberia (REDRESS Consortium, 2025). The programme uses a person-centred approach to evaluate, develop and adapt health systems interventions (including human resource management (HRM)) for the care of persons affected by skin NTDs in Liberia and beyond.

We conducted a review of literature to identify strategies to provide support and manage health workers in caring for people with NTDs in Low- and Middle-Income Countries (LMIC) (REDRESS Consortium, and Ministry of Health Liberia, 2023). Key strategies included: 1) the importance of monetary and non-monetary incentives (Awah and Kum, 2018, Krentel et al., 2017); 2) the role of gender in selection and support of health workers and CHWs at all levels (Oluwole et al., 2019, Vouking et al., 2015); 3) the importance of health systems recognition (Awah and Kum, 2018, Dadun et al., 2016, Oluwole et al., 2019) and 4) community support (Dadun et al., 2016, Omer et al., 2020). Within our study we have sought to explore human resource management (HRM), which we define as “a strategic, integrated and coherent approach to the employment, development and well-being of the people working in organisations" (page 5; Armstrong and Taylor, 2010). The review we conducted showed there is literature on the application of individual HRM practices for health workers delivering NTD services, however, there is little evidence on a coordinated HRM approach to support health workers, whereby HRM practices are designed to not only address expectations, but also ensure that the NTD programme meets its goals (Raven et al., 2015). In addition, none of the papers reviewed focused on Liberia, where an integrated approach to managing NTDs has been adopted. In this paper, we explore the context of HRM relating to skin NTDs, from the perspectives of stakeholders across health systems levels with a focus on performance management perspectives, in order to identify a bundle of interventions that focus on enabling health workers to perform their roles in delivering care for people with skin NTDs.

## Materials and methods

### Study Design

This study used a naturalistic paradigm seeking to give emphasis and to understand the experiences and views of both voluntary and salaried health workers from community to national level (Malterud, 2001), relating to their experiences and motivations caring for people affected by skin NTDs. Qualitative (key informant and in-depth interviews and focus group discussions) and participatory research (photovoice) approaches were used because they help to understand the experiences, meanings and views of health workers within their natural settings, giving due emphasis to the meanings, experiences, and views of all the participants (Pope and Mays, 1995).

#### Study context

**Fig. 1:**
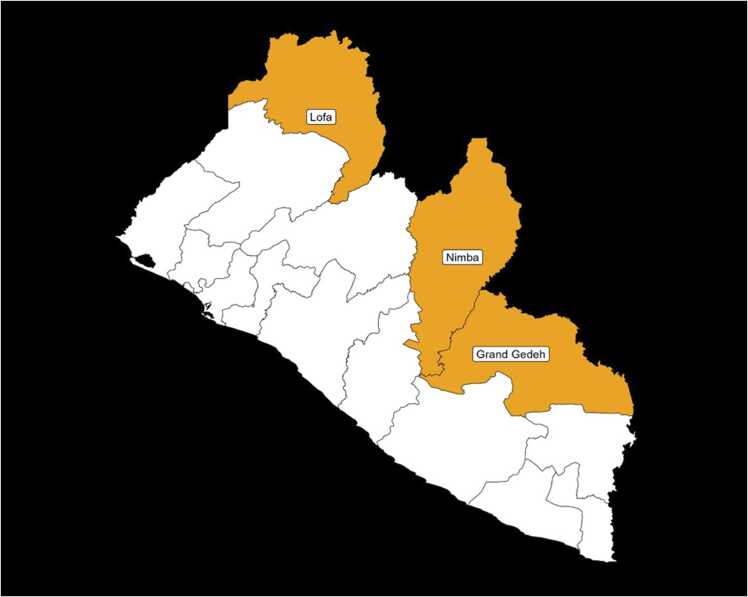
Map of Liberia showing the three study counties.

The findings presented in this paper were collected as part of a formative research study including a context analysis of existing integrated approaches to the management of skin NTDs carried out between November and December 2020. Primary data were collected in three counties (see Fig. 1) including:

• Lofa County, in the Northern part of Liberia was selected because integrated (within the health system and between diseases) case management of skin NTDs was rolled out within this county in 2016;
• Grand Gedeh County, in South-Eastern Liberia was selected as no case management of skin NTDs has been rolled out within this county;
• Nimba County was selected as Ganta Hospital, a national referral hospital for skin NTDs, is located here. Therefore a smaller number of key informant interviews were carried out with health workers with unique specialist experience in caring for patients with skin NTDs at this referral hospital, who are subject matter experts at this tertiary referral hospital. Additional interviews at other health systems levels were not carried out in Nimba County since it was not selected for additional intervention.

Within Lofa and Grand Gedeh Counties, data were collected from two (out of six) purposively selected (based on geography and population) districts within each County.

#### Participant selection and sampling method

Participants included health workers and managers from all levels of the health system (community, primary health care facility, secondary and tertiary hospitals, district, county and national levels) to enable a holistic exploration of current HRM practices (Table 1). Participants were purposively sampled based on their experience either providing care for people affected, or in the planning of services for people affected by skin NTDs until saturation was reached. Participants were recruited by researchers from UL-PIRE (Liberian research organization) in collaboration with the County Health Teams (CHTs).

**Table 1:** Participant demographics.

**Category of participant**	**Number national level**	**Number Lofa County**	**Number Grand Gedeh County**	**Number Nimba County**	**Total participants**
Key Informant Interviews					
National level	16 (0F, 16M)				16 (100 %M)
County health team level		14 (4F, 10M)	13 (1F, 12M)	0	27 (19 %F, 81 %M)
County referral hospital level		3 (2F, 1M)	2 (2F, 0M)	0	5 (80 %F, 20 %M)
In depth Interviews					
District level		5 (0F, 5M)	4 (1F, 3M)	0	9 (11 %F, 89 %M)
Health Facility level (officer in charge (OIC), second Screener surveillance officer, lab tech, dispenser/pharmacist)		5 (1F, 4M)	7 (3F, 4M)	3 (0F, 3M)	15 (27 %F, 73 %M)
Community Health Services Supervisors (CHSS)		2 (1F, 1M)	2 (2F, 0M)		4 (75 %F, 25 %M)
Community Health Assistants		2 (0F, 2M)	2 (0F, 2M)		4 (100 %M)
Community Health Promoters		2 (0F, 2M)	2 (0F, 2M)		4 (100 %M)
Focus Group Discussions					
District level		1 FGD (1F, 2M)	1 FGD (1F, 3M)		7 (29 %F, 71 %M)
Health Facility level		1 FGD (3F, 3M)	1 FGD (2F, 3M)		11 (45 %F, 55 %M)
Photovoice					
CHAs		4 (2F, 2M)	4 (0F, 4M)		8 (25 %F, 75 %M)
CHPs		4 (2F, 2M)	3 (1F, 2M)		7 (43 %F, 57 %M)

F = female; M = male.

The methods and additional findings from the photovoice study are described more fully elsewhere (REDRESS Consortium, 2022).

### Conceptual framework

**Fig. 2:**
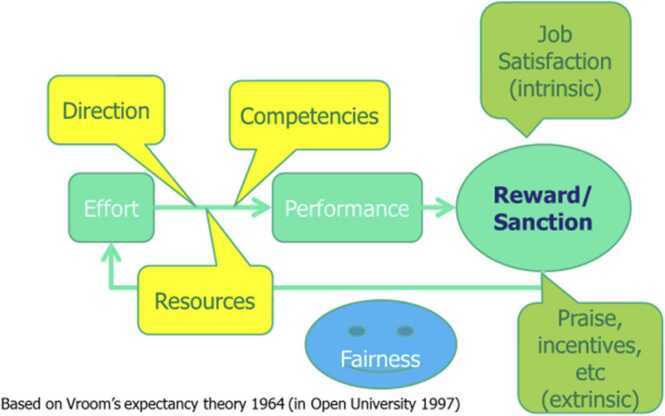
Vrooms expectancy theory.

Our study design, data collection and analysis were shaped by Vroom’s expectancy theory, which states that performance is influenced by the direction, competencies, resources and rewards available to health workers (Vroom, 1964). This in turn influences the worker’s level of effort which they put into their work and their performance (see Fig. 2). This framing was used to guide the development of topic guides, to explore these four influencing factors with a focus on performance management.

#### Data collection and analysis

Within each county, research coordinators (WT, HB, JK, JSS, CP, ZZ) provided training, mentorship and support to two to three co-researchers (Community Health Assistants (CHAs), Community Health Services Supervisors (CHSS) and persons affected by skin NTDs) who were recruited from within each county. The co-researchers played a vital role in guiding the researchers through their lived experience as health workers and as persons affected. Research was supported remotely by a team of researchers from Liverpool School of Tropical Medicine (JR, LD, ST, RM) and Effect Hope (MP).

Key informant interviews were carried out from November to December 2020 with 16 national key informants and 32 county key informants from Lofa and Grand Gedeh Counties (including NTD focal persons, community health focal persons, mental health focal person, county pharmacist, county data management staff, diagnostic and surveillance officers) to explore their experiences with human resource management practices. 36 in-depth interviews were carried out with health workers including officer-incharge (health facility manager, who maybe a nurse or clinical officer), second screener (health facility second in charge, usually a nurse), dispenser, laboratory technician, community health service supervisor (a clinically trained health worker, usually a nurse, responsible for supervising CHAs and CHPs), community health assistant (CHA) (a community health worker living greater than 5 km from the closest health facility who receives regular stipend) and community health promoters (CHP) (a volunteer community health worker living less than 5 km from the closest health facility) to explore their experiences with providing care for persons affected by skin NTDs, and their experiences and views of current HRM practices. This included questions which prompted reflection on care provided relating to mental health, since this had been identified by Ministry of Health as a priority area for strengthening person-centred care for persons affected by skin NTDs. FGDs were conducted with health workers from district and health facility levels in each county to explore similar topics as the in-depth interviews, but using the group interactions to generate further discussion on the topic (Pope and Mays, 1995). Interviews and FGDs were carried out at a time and place convenient to the participants. Photovoice is a collective visual method, originating from a community-based participatory research approach using photographs to represent issues which are important to those involved (Ronzi et al., 2019). Photovoice was undertaken over a period of four weeks in November 2020 with CHAs and CHPs to enable them to tell their everyday stories through pictures (see ethics section for training details). Participants were asked to take photos of their work, work environment and motivators with a particular focus on skin NTDs. The photovoice process is summarized in Box 1 below (adapted from (Ronzi et al., 2019).

Box 1 **Steps for Photovoice process, adapted fromRonzi et al. (2019)**

*Step 1: Select and recruit the target audience of photo voice participants (CHAs and CHPs)*

*Step 2: Introduce the photo voice methodology to participants and informed consent*

*Step 3: Participants camera training – participants trained about ethics and informed consent and asked to take photos of their work and interactions with the health system.*

*Step 4: Weekly reflective meetings – discuss photos with researcher during one on one meeting*

*Step 5: Camera collection and photo discussion*

*Step 6. Photo categorization – photos discussed as a group and themes identified*

*Step 7: Photo dissemination and analysis workshop*

All data collected were audio recorded, transcribed verbatim and stored to SharePoint. A selection of approximately 10 % were randomly selected, with the transcript cross-checked against the audio for quality assurance purposes.

Thematic framework analysis was carried out in order to help classify and organize data according to the key themes, concepts and emerging categories (Ritchie et al., 2003), where the researchers familiarized themselves with the data before discussing jointly the main issues within the transcripts in order to jointly develop a coding framework, generated inductively from the transcripts (WT, RM, JR, HB, JK, JSS, CP, ZZ, LD). This coding framework was then applied to the data using NVIVO12 to help data management as part of analysis. After coding of the data, charts were developed, followed by developing themes and drafting narratives to develop preliminary reports, comparing and contrasting between types of health workers and between counties (Ritchie et al., 2003). Findings were reviewed and grouped according to our theoretical framework (direction, competency, resources, satisfaction)(Vroom, 1964).

Data findings were shared with a wide range of stakeholders (county decision makers, informal providers, health workers across health systems levels, community leaders and persons affected by skin NTDs) in dissemination workshops (September 2021) which were used to prompt discussion and identify a range of interventions to strengthen holistic care for persons affected by skin NTDs, including a bundle of interventions to strengthen HRM. Following the initial county dissemination meetings, an HRM technical working group met regularly online to progress the development of the intervention bundle components, before these were packaged within the MOH Liberia integrated case management intervention manual (REDRESS Consortium and Ministry of Health Liberia 2020). This bundle was later rolled out and evaluated during a 12- month intervention during October 2022 – September 2023 (paper forthcoming).

## Results

In this section, we use Vroom’s expectancy theory to present our findings. We provide details on the current practices in terms of providing **direction** to health workers including job description, roles and responsibilities, supervision and appraisal; enabling health workers to have the **competencies** to perform their responsibilities through training; ensuring adequate **resources** are available to health workers including salary, drugs and supplies and provisions for supervision; and the **rewards** that they receive for their efforts, including intrinsic and extrinsic motivators. These are summarized in Table 2. Headings in grey within Table 2 are based on Vroom’s theory, sub-headings emerged from the findings.

**Table 2:** Summary of HRM findings and recommendations.

	**Findings**	**Recommendation**
Direction		
Job description	Infrequently described by all cadres.	Add NTDs within health management information system (HMIS) to support supervision. Make available needed funding, resources, and forms for supervision. Introduce performance appraisal and HRM approaches to provide more guidance for health workers. Link well health workers for peer mentoring.
Roles and responsibilities	Limited role relating to care of NTD patients among facility health workers (especially in Grand Gedeh). Generally clear awareness of NTD-related roles, but no discussion of role in supporting mental health for persons affected by skin NTDs.	
Supervision	Source of motivation. Provides opportunity for mentoring. Limited training or funding for supervision and support, resources. No supervision of mental health care.	
Appraisal	Limited discussion.	
Competencies		
Knowledge of skin NTDs	Lack of knowledge hinders care. Limited awareness of case management in Grand Gedeh.	Expand roll out of training. Develop tools to support application of learning, e.g. NTD manual, algorithm.
Training	Donor dependent. Trainings often too short to embed knowledge. High demand for trainings. Promotes trust between health workers and community.	
Resources		
Salary	Many facility workers work on a voluntary basis. CHPs do not receive regular payment. Attendance sporadic in the absence of salary.	Provide regular timely salary. Provide accommodation for health workers. Ensure availability of drugs and supplies, rain gear, fuel for supervision and surveillance activities.
Drugs and supplies	Need for consistent supply chain emphasised in Lofa County. Lack of drugs and supplies is a demotivator for health workers, and compromises trust with patients.	
Resources for supervision and surveillance	Lack of needed resources for CHAs and CHPs to do work, e.g. raingear. Lack of fuel, laptop, motorbike for supervision.	
Rewards		
Intrinsic motivation	Health workers have strong responsibility to patients, often caring beyond assigned responsibilities.	Ensure access to basic social services for health workers, e.g. internet, electricity, accommodation, banking facilities, timely salary, telecommunication, road infrastructure, suitable schools for their children.
Extrinsic motivation	Discontent about many facility staff not on payroll.	

### Direction

The discussion about direction included four main aspects: awareness of job description, roles and responsibilities, supervision, and appraisal.

#### Job description

Health worker job descriptions were infrequently described by facility and community level respondents, although they were discussed by a small number of supervisors who described carrying out supervision in accordance with their supervisee’s job description.

#### Current roles and responsibilities

**Photo 1:**
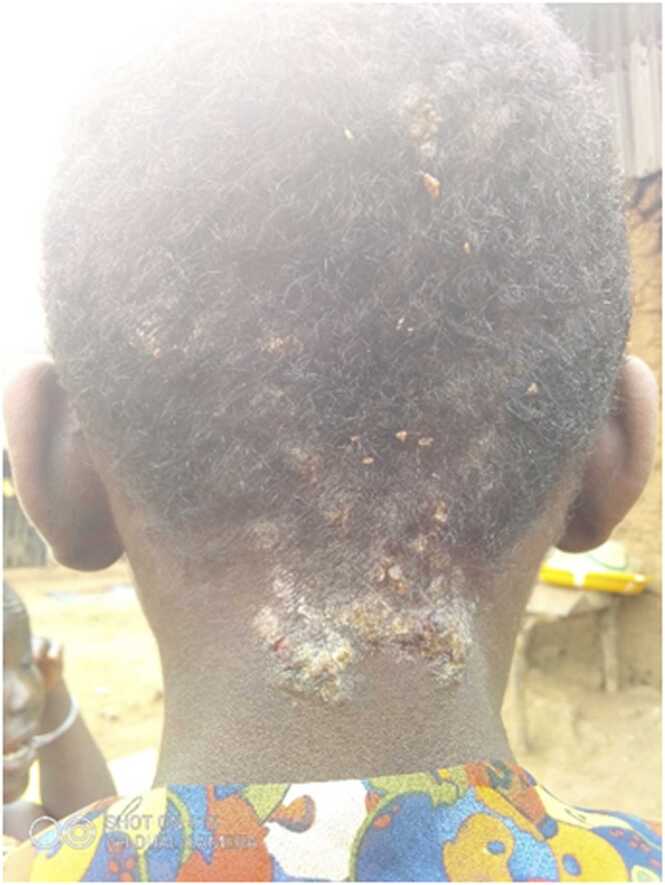
Skin condition on back of head.

Health workers gave clear descriptions of their role and responsibilities; however, surprisingly facility health workers rarely discussed roles relating to the care and management of persons affected by skin NTDs (especially for Grand Gedeh County where integrated case management of skin NTDs had not yet rolled out). There was not felt to be much gender-related influence on health worker roles related to caring for patients with NTDs, with the exception of hydrocele patients, who preferred to consult with a male health worker. CHAs and CHPs from Lofa and Nimba counties gave clear descriptions of job roles relating to raising awareness of skin NTDs, surveillance, identifying and referring persons affected by skin NTDs (see Photo 1).

*“I came across this condition [*Photo 1*] while we were keeping surveillance. And when you are keeping surveillance, you do not only look for what you are told to but to include any other problem that you think is a health issue.” Male CHA photovoice participant, Lofa County.*

No health worker described the provision of mental health support as part of their role for persons affected by skin NTDs. However, several key informants (mainly from county and national levels) described that people with chronic conditions (including skin NTDs), should be supported both physically and mentally. Meanwhile, compassion for persons affected, including recognition of their mental and social wellbeing, was a theme identified by CHA and CHP photovoice participants.

Excessive workload was described by national level respondents as a challenge for facility health workers in remote areas, contributing to frustration. Additionally, there was felt to be a high burden placed on the NTD focal person who was responsible for all NTD cases, creating a bottleneck with care if he was unavailable.

*“They (health workers) might be overwhelmed and at the end of the day, you get frustrated and sometime do not approach patients the right way. So, all those stuffs have to do with performance.” Male National Key Informant 19*

#### Supervision

**Photo 2:**
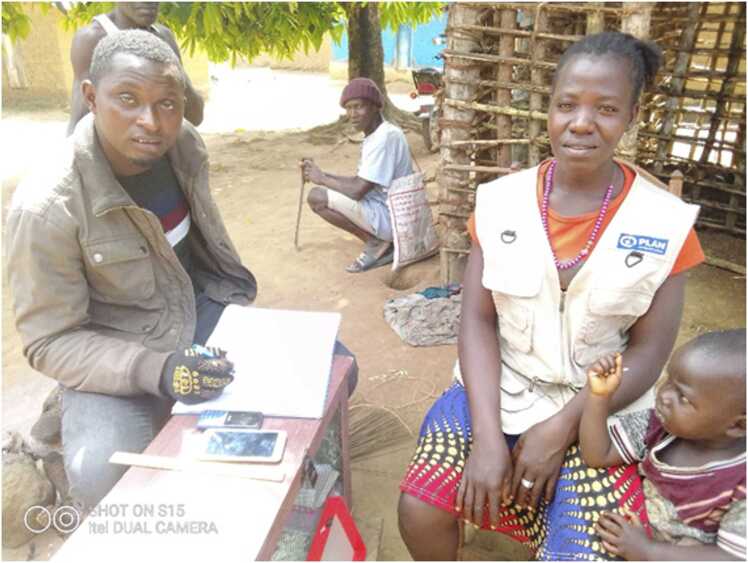
CHA with supervisor.

Supervision was a source of motivation for a range of health workers, as well as providing opportunity for mentoring (see Photo 2). The main supervision methods include: Joint Integrated Supportive Supervision (JISS) (a standardised MOH supervision checklist which covers a wide range of conditions, including NTDs carried out at the health facility level), which was often the primary supervision method, NTD supervision from national to county level, mentoring of staff and informal supervision.

*“In this photo I am happy for my supervisor to visit me, to correct me. Any mistake he will let me know so the next visit I can make that mistake again. So, I am very happy."* Female CHA Photovoice participant, Lofa County.

There was felt to be a disheartening gap in needed resources and needed training (motorbike, fuel, funds, training) for the delivery of supervision from county and district level staff to health workers at facility level for NTD care and mental health, particularly in Grand Gedeh. Supervision of CHAs by the CHSS was an established supervision method. Meanwhile, there was no support for mental health supervision described in any county, and no established link between the NTD and mental health programmes.

*“We that are [doing the] supervising, we are not trained [how to supervise], then how about people we are supervising?” Grand Gedeh District level Male KII 026*

#### Appraisal

IDI participants (mainly those at county level) suggested that introducing performance appraisal, patient feedback and review meetings, and adoption of HRM approaches for poor performance, e.g. written warning would provide opportunities to provide more guidance and direction for health workers and promote improved performance.

Recommendations to strengthen direction included: adding NTDs within the HMIS form to support supervision of staff, with adequate availability of reporting forms for health workers. Examples of peer mentoring between hospital workers, pairing well performing and weak performing health workers for mentorship support were provided. The need for stronger outward facing communication, especially from the laboratory was described as important for health workers to have the needed understanding about diagnosis to manage their patients appropriately.

*“So, for recommendation, for NTD we are working together but we need communication from the focal person to the laboratory staffs … I am the supervisor, I am talking to them they are doing their work. They should try to motivate them.” Grand Gedeh County level Male KII008*

## Competencies

### Knowledge of skin NTDs

Due to limited access to laboratory testing, clinical judgement was described as critical in informing diagnosis and care. Where clinicians do not have the needed knowledge, unnecessary referral, or even misdiagnosis was described. Health workers described being motivated to care for persons affected but that this was hindered by their lack of knowledge.

*“For me I have the passion to do the work, but I don’t have enough knowledge to do the work.” Grand Gedeh Health facility Male IDI 060*

Much of the case management knowledge was felt to sit with the NTD focal person within the county, with knowledge gaps relating to case management at facility and district levels. This contributed to bottlenecks in the care pathway, with delayed diagnosis and management.

*“But is a major gap the focal person will have to be running and is not possible for him to cover the whole county. … it becomes a serious concern. To bridge that gap, like I said, fill in the knowledge gap because of NTDs; not many of our clinicians have knowledge on NTDs.” Grand Gedeh County Male KII 022*

Knowledge of the implementation of the integrated case management NTDs plan, including understanding diagnosis and tracking of NTD cases by health workers at facility and community levels was common among county, district, and community level personnel in Lofa and Nimba Counties. In contrast, health personnel were less aware of the plan in Grand Gedeh County where only basic components of the plan were passively implemented at the time of data collection, and where only one officer in charge at just one facility per district in Grand Gedeh County had basic knowledge about case management.

Knowledge gaps were described by most managers and clinicians including: identification, diagnosis, management and reporting of skin NTDs. This was frequently described as needed by almost all participant groups (and most strongly expressed by surveillance, M&E and laboratory staff). Where this was described, it was usually followed by recommendations on the need to train and capacitate health workers across the county.

### Training

Participants reported that training of health workers is often donor funded. Since NTDs are often not a priority for major donors, this has resulted in limited funds to carry out the needed trainings with some health workers never having been trained about skin NTDs. Where trainings do take place, they were described as being too brief, with inadequate time to embed the needed skills, especially for reporting.

Health workers at facility level from across all counties (and in both FGDs and IDIs) recommended that they receive training in order to provide them with the knowledge to enable them to fulfil their roles in caring for persons with skin NTDs, and in completing ledgers and reports. Training was also felt to promote trust between the health workers (at facility and community levels) and the community due to the perception of improved skills of the health workers following training. It was also felt to bring motivation through increased capacity and ‘self-actualisation’.

*“Training builds people capacities, training helps to motivate people, it also promotes the self-actualisation of the personnel, and in the context that no knowledge is wasted and that can help”. Male National KII 07*

Turnover of health workers previously trained in Lofa county created knowledge and skills gaps, which the NTD focal person attempted to mediate through mentorship during supervision visits.

*“Since I have been to this health centre, not one day I have gone under training that I am told this is a skin disease or to discuss it.” Lofa District Level Mixed FGD 02*

Recommendations to strengthen competencies included: More comprehensive training for CHAs and CHPs, surveillance, M&E, and laboratory staff was emphasised in interviews and FGDs. Training was also felt to be a source of motivation due to the daily subsistence allowance (DSA) provided for attending trainings (particularly in the absence of regular or inadequate salary).

The availability of tools to support application of learning was emphasised, with recommendations for NTD manual, an algorithm to guide health workers towards diagnosis and reporting tools and more practical within historically highly didactic training processes. Working towards greater future sustainability, the inclusion of case management within pre-service training was described by one county level respondent.

*“Another recommendation is … the integration of NTDs in the pre-services at the different schools, you understand.” Grand Gedeh County Level Male KII 022*

### Resources

Respondents described the necessity for provision of a timely salary, the availability of needed materials for caring for patients, including drugs and supplies, as well as materials for effective supervision as essential to both motivation of health workers and to enable quality patient care.

### Salary

National and County Level health workers felt that clinical health workers are not adequately compensated, with many facility level health staff working on a voluntary basis. Likewise, at community level CHAs are supposed to receive a regular monthly payment since 2017 introduced as part of the Ministry of Health’s revised community health policy. However, CHPs (community health workers who live less than 5 km from a facility) do not receive any form of regular incentive or compensation. In the absence of regular government salary, attendance at work (across health systems levels) was described (and accepted by supervisors) as sporadic, since health workers must seek alternative ways to earn a salary to support their family.

*“Yes, mainly the salary issue, is making some of our staffs that have worked over two years, almost two years they can’t get on pay, they are very weak in coming to work.” Grand Gedeh Health Facility Female IDI 015*

### Availability of drugs and supplies

Availability of drugs and supplies was discussed more frequently among participants from Lofa County, since this was where integrated (within the health system and between diseases) case management of skin NTDs was rolled out. Within Lofa county the need for a consistent supply chain, with availability of needed drugs and supplies to care for patients was emphasised.

*“Having the knowledge is not all about it, but the need for support that has to do with the drugs and other things to handle these cases, makes the work effective”. Lofa District Level Mixed Gender FGD 001*

The lack of drugs and supplies at facilities was a demotivator for health workers (contributing to embarrassment and frustration) and compromised trust among community members, which both IDI and FGD participants felt threatened early care-seeking by people affected by skin NTDs.

*“Yes, it’s like you will just become frustrated, the patient is frustrated, and you the clinician is frustrated because you have seen a case that you know you can manage, but don’t have drugs and all of those things.” Lofa County Level Male KII011*

#### Availability of resources for supervision and surveillance

Where resources for supervision were available, they were frequently insufficient and provided through donor donations rather than from the county health budget, contributing to delays in supervision activities. In contrast, national level participants did not perceive a gap in availability of needed resources for supervision, describing having previously provided resources for supervision which include: laptops, motorbike (for NTD focal person in pilot counties), communication cards and fuel for supervision.

**Photo 3:**
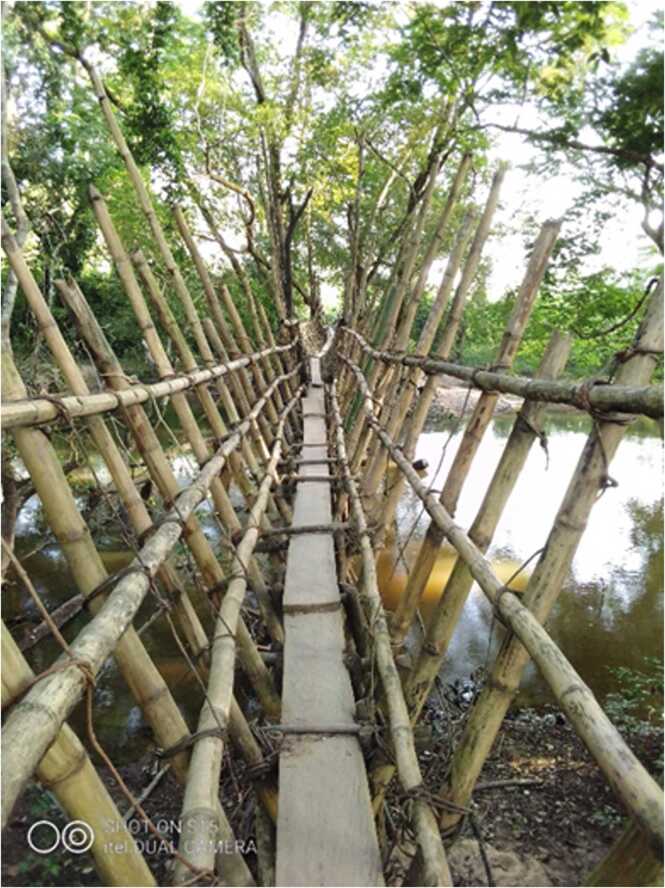
'Monkey bridge' leading to remote community*.*

County level respondents described the resource gaps for surveillance as including a lack of financial support, damaged bicycles, shortage of fuel, lack of rain gear for surveillance staff (CHA, CHP, CHSS, DSO) and no transportation or phones for active surveillance teams. This created barriers to effective surveillance activities. CHAs and CHPs also described the challenges reaching some of the more remote communities through the photovoice study (see Photo 3).

*“So I call this photo as a challenge.… And I was on my way to the community and this monkey bridge is not well prepared. Sometimes you are walking in it, it can be shaking. Sometimes how the ropes looking are not really guarantee. So I took this photo to like you know that my very self I walk in this monkey bridge to get to the community.”* Male CHA photovoice participant, Lofa County.

Recommendations to strengthen resources included: providing regular and timely government salary for health workers, making accommodation available for health workers, ensuring availability of needed drugs and supplies, providing rain gear for surveillance staff, and supplying fuel for supervision and surveillance activities.

## Rewards

### Intrinsic motivation

Health workers frequently have a strong sense of responsibility to care for their patients, with some describing acting beyond their assigned responsibilities, including buying drugs or food for patients using their personal money. Intrinsic motivators were most commonly described by health workers at facility level and by CHAs and CHPs. There was a sense of responsibility and obligation from many facility health workers that they must continue to work, regardless of whether they receive a salary or not. A small number of national managers felt that health workers based in their hometown, are motivated to serve their own relatives (even without a salary). With CHAs and CHPs expressing their desire to help their community.

*“You can’t abandon work because there is no money.” Lofa Health Facility Health worker Male IDI 012*

### Extrinsic (de) motivation

In general, extrinsic motivation often seems to have been understood as referring to compensation (either salary or another form). There was widespread discontent relating to health workers not having been added to government payroll or where no salary increment had been added. This has impacted performance negatively (see resources section).

*“The officer in charge (OIC) that supposed to be at the facility is being under paid… so he has to leave the facility, come and give us excuse … he will want to move from there and come run after it because he has family to attend to.” Grand Gedeh District Level Mixed Gender FGD 003*

In the absence of a government salary, health workers often receive rice by way of encouragement, and their name is recommended for recruitment, whenever there is a new salaried position. FGD participants described being added to payroll as a gradual process. Additionally, key informants described performance-based financial and non-financial incentives in place at district, facility and individual levels. These are mainly provided for performance relating to conditions other than NTDs. Health workers in FGDs also described being motivated by recognition from their community.

Recommendations for motivation: One national level manager said the government needs to create a more conducive environment for health workers by ensuring access to basic social services including internet, electricity, accommodation, banking facilities, timely remuneration, telecommunication roads, and schools for their children. Other recommendations to motivate health workers include: promotions, recognition and study opportunities.

### Performance

National KIIs generally perceived performance as lower than expected. When performance was discussed, the factors most commonly identified as influencing it were: lack of salary; lack of social services available in remote locations; lack of needed drugs and supplies to deliver care; excessive workload in remote locations; and unclear direction. These were felt to relate to decentralisation of some, but not all aspects of health services, with HRM still centrally managed, and HRM procedures relating to annual leave not implemented according to policy leading to lack of clarity. Strategies identified by national KIIs to manage performance include those already described within this paper (training, supervision, regular salary, strong supply chain).

## Discussion

This is one of the first studies to document the specific roles of the health workforce in relation to the integrated management of skin NTDs and, to the best of our knowledge, the first study to consider implications for broader health systems HRM using Vroom’s expectancy theory framing. Within the discussion, we revisit findings and recommendations from our study in light of global literature. Health workers in Liberia often have strong intrinsic motivation to care for people affected by skin NTDs, however, this is undermined by weak HRM structures particularly in areas where integrated services for NTDs requiring case management have not yet been rolled out, including: limited awareness of NTD role; gaps in knowledge and skills (for both NTDs and for mental health); irregular supervision; and limited resources needed to deliver care. A key identified gap was the ability of health workers to provide mental health support. Alongside the role of health workers our study also explored the role of faith and traditional healers in caring for people with skin NTDs, which has been presented elsewhere (Berrian et al., forthcoming). It is anticipated that a bundle of HRM approaches are needed to strengthen performance of health workers caring for patients with skin NTDs.

### Direction

CHAs and CHPs clearly described roles in raising awareness, identifying and referring people with a possible NTD, however, none described having a role in providing psychosocial assessment for people affected, despite this being included within the CHA training module. The lack of clarity surrounding roles and responsibilities for community health workers in other settings providing NTD service delivery has previously been described by Omedo et al. (2012), who described confusion among CHWs relating to responsibilities for sample collection and drug distribution in Western Kenya. However, most of this literature has explored CHW role relating to mass drug administration. This is the first study (to our knowledge) to explore roles for both CHWs and skilled health workers for integrated management of persons affected by skin NTDs.

Facility level health workers identified job aids which they felt would provide guidance and direction when caring for NTD patients, including NTD manual, clinical algorithm and reporting tools. Similar approaches have been described elsewhere in the literature, including within the WHO algorithm as part of the ‘Recognizing neglected tropical diseases through changes on the skin: a training guide for frontline health workers’ manual and skin app (WHO, 2018), but their impact for the care provided by health workers remains largely undetermined (Akoko et al., 2019, Santmyire, 2016, Taal et al., 2015). There are some challenges to be aware of when introducing new guidelines and tools, including health workers being unaware of the guidance (Amgarth-duff et al., 2019), tools being difficult to follow or not available in the local language (Oluwole et al., 2019), or the application of guidelines being undermined due to a lack of needed resources to follow them (Amgarth-duff et al., 2019). Therefore, when developing guidance as part of intervention development, the need to include pictorial images, with simple language that are easy to follow, and which have been piloted and adapted with those health workers who will use them is important (Oluwole et al., 2019, Taal et al., 2015).

Participants in our study described supervision as motivating, in keeping with global studies where it was generally viewed positively with improved motivation, confidence, skills development (Katabarwa et al., 2010, Krentel et al., 2017, Silumbwe et al., 2017, Stanton et al., 2015). Positively, we found that NTD supervision structures were described within existing MOH structures, in contrast to systematic review in Sub Saharan Africa by Vouking et al. who found parallel NGO structures (Vouking, Violette Claire Tamo, 2013). Despite clear structures, barriers to supervision existed, as a result of limited funding and fuel to travel to facilities during supervision. Opportunities for mobile supervision via WhatsApp platforms has potential to temporarily bridge these gaps (Akoko et al., 2019, Stanton et al., 2015). However, care must be taken to ensure health workers feel adequately supported as studies have found that where NTD health workers do not receive supervision they may feel demotivated, alone and unsupported (Krentel et al., 2017, Oluwole et al., 2019).

### Competencies

In keeping with our study, where knowledge and training were among the most frequently described mechanisms to improve health workforce management, there have been multiple studies globally exploring knowledge and training about NTDs for health workers (Acup et al., 2017, Kawuma et al., 2011, Krentel et al., 2017, Omedo et al., 2012, Taieb et al., 2018). We found knowledge gaps in both counties, with health workers having never been trained in case management in Grand Gedeh County with no refresher training in Lofa County. In keeping with literature, the knowledge gaps resulting from lack of training have implications for health worker satisfaction, as well as patient care, impacting health workers’ ability to fulfil tasks correctly, such as how to identify or diagnose a patient with an NTD (Acup et al., 2017), or how to treat a patient (Amgarth-duff et al., 2019; Kawuma et al., 2011; Oluwole et al., 2019, Taieb et al., 2018). We found that well trained health workers built trust with the community, similar to a study by Krentel et al. (2017) who found that inadequate training which is too short/ lacking in content, can hinder performance and contribute to mistrust of the community towards volunteers (Krentel et al., 2017).

### Resources

Our study found that the lack of adequate incentives for working in remote locations created challenges for health workers who worked there. This is in keeping with Duamor et al. (2017) who found that staff in South Western Cameroon avoided working in hard-to-reach communities to provide mass drug administration, with equity implications for local community (Duamor et al., 2017).

Our findings that health workers frequently do not have the resources needed to manage patients appropriately, including drugs and supplies, has been widely described elsewhere in NTD global studies (Duamor et al., 2017, Fleming et al., 2016, Oluwole et al., 2019, Santmyire, 2016, Shetty, Vanaja et al., 2012). Globally, the literature reveals similar supply chain challenges impacting on health workforce performance, including limited transportation for drugs (Duamor et al., 2017, Oluwole et al., 2019), inadequate forecasting of quantities of drugs and supplies needed (Fleming et al., 2016, Oluwole et al., 2019) and inappropriate drug delivery strategies at both country and community levels (Silumbwe et al., 2017). The reasons for NTD supply chain challenges in Liberia were not explored in this study, but have been described by Kollie et al. (2023) and include: reliance on donor funding to procure drugs; difficulty accessing commodities due to bureaucratic bottlenecks; poor coordination and exclusion of NTD commodities from electronic data tools (Kollie et al., 2023).

### Rewards

Our study found high intrinsic motivation among health workers to care for NTD patients, despite the series of HRM challenges described. Participants identified a range of options to improve motivation among health workers. Within the literature community support was a strong extrinsic motivator for many health workers caring for patients with NTDs, especially among community drug distributors providing services for their own relatives (Katabarwa et al., 2010), and among community volunteers for NTD programming in Haiti who had very strong intrinsic motivation (despite late or absent pay, very limited programmatic support, limited resources, etc.) that was frustrated by extrinsic and management-related programmatic factors (Wodnik et al., 2020). Recognition from the community, pride in providing a service and positive feedback from the community were all key motivators (Krentel et al., 2017, Oluwole et al., 2019). When health workers are not able to provide this care, this can lead to moral dilemmas and distress, limiting progress towards the delivery of person-centred systems (Dean et al., 2023).

There were limited findings in our study of the performance of men compared with women. Krentel et al. (2017) found that few studies consider the effect of gender on NTD volunteer performance. Previous literature has revealed mixed findings with some indicating that women were more likely to be perceived as committed and patient (Vouking et al., 2015), while another felt men were more active than women, but may be more impatient (Fleming et al., 2016).

### Mental health support

No health worker described having a role in providing mental health support for persons affected by skin NTDs. Additionally, there were no resources provided to support mental health related supervision. The need to provide mental health support for persons affected by skin NTDs has been highlighted within the WHO NTD Roadmap, with a programme priority being the ‘development of training materials for health workers with emphasis on integrated pathways for…mental health, reduction of stigmatization and discrimination and psychosocial support’ (page 48 (World Health Organization, 2022)). This study worked to develop a bundle of interventions which sought to support health workers to provide mental health support for persons affected by NTDs across levels of the health system from population, to family and community, decentralised level and finally referral to secondary and tertiary care. This included developing a range of training materials, adapting job tasks to reflect mental health roles, training CHAs and CHPs in look listen link for mental health support, training health workers in integrated care including screening and provision of basic psychosocial support for common mental health conditions, including record keeping for mental health indicators integrated into the health management information system for NTDs, training health workers in mhGAP (mental health gap action programme), and providing joint supportive supervision including mental health, community health and NTD supervisors.

### Holistic human resource management needed for strengthening performance

**Fig. 3:**
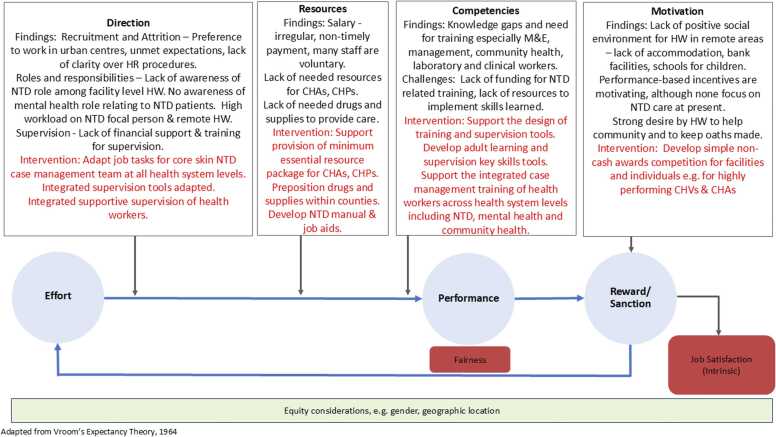
Revised HRM intervention pathway.

Based on our findings, and previous studies which have revealed the value of complementary strategies which address performance (Raven et al., 2015), we co-developed (together with health decision makers and workers from community through to national health systems levels, persons affected and informal providers) a bundle of interventions to strengthen HRM for health workers across health systems levels caring for persons with skin NTDs, with the aim of improving performance. Many of our findings, and the bundle of interventions developed resonate with a review by Kok et al. (2014), which highlights the importance of a mix of incentives, regular supervision and refresher training, and strong community-health system links (Kok et al., 2014). We developed an intervention pathway linked to Vroom’s theory and informed by our findings that both identified challenges and suggestions for improvement, reflecting our findings (black text) as well as identified avenues for enhancing performance (red text), which were used as the basis for developing an HRM package as part of REDRESS intervention (see Fig. 3). The next step was to implement this package and evaluate its effect on health worker performance and skin NTD service delivery and outcomes (publication forthcoming).

### Study strengths and limitations

Travel restrictions due to the COVID 19 pandemic resulted in remote working for extended time periods. This enabled leadership within the Liberian research team, supported through technical and training support provided online by LSTM team (LD, RM, ST, JR). Additional limitations include that a majority of participants included in the study are male. While efforts to include both men and women were made during selection of respondents, many of those included, particularly at senior levels, were men. This relates to many of the senior positions at national and county levels being occupied by men. This is in keeping with findings from the ‘Gaining Ground?’ 2024 report, which finds that systematic reform is needed to bring about diverse and inclusive global health governance (Global 50/50 Towards gender equality 2024). Additionally, selection and recruitment of health workers at lower levels was facilitated by county level health workers, who often identified men as participants. In order to promote trustworthiness within this study, co-researchers were involved within the research team to facilitate community entry and to support participants to be able to express themselves freely promoting credibility. Data was triangulated across different types of participants, locations and methodologies to promote credibility of data. We reached saturation through the interviews promoting dependability, and meaning was checked with participants at the end of the interviews to promote confirmability (Guba, 1981).

## Conclusion

The quality of neglected tropical disease (NTD) service delivery relates closely with the work carried out by health workers, both skilled and voluntary. While many health workers are motivated to care for their patients, we found that they were not adequately supported by existing HRM structures, impacting care for people affected by skin NTDs. These gaps in health workforce management have implications for the equity of NTD care for persons affected, compromising Liberia’s ability to work towards achieving WHO Roadmap 2030 targets (World Health Organization, 2022) and to attaining Universal Health Coverage. In response, as part of the REDRESS intervention development process, we developed a bundle of HRM interventions to strengthen and promote performance of health workers, to equip and motivate them to provide person-centred care for persons affected by skin NTDs.

## CRediT authorship contribution statement

**Laura Dean:** Writing – review & editing, Supervision, Resources, Methodology, Investigation, Formal analysis, Data curation, Conceptualization. **Zeela Zaizay:** Writing – review & editing, Supervision, Project administration, Investigation, Formal analysis, Data curation. **John S Smith:** Writing – review & editing, Methodology, Investigation, Formal analysis, Data curation. **Emerson Rogers:** Writing – review & editing, Supervision, Project administration. **Karsor Kollie:** Writing – review & editing, Supervision, Resources, Project administration. **Colleen Parker:** Writing – review & editing, Methodology, Investigation, Formal analysis, Data curation. **Maneesh Phillip:** Writing – review & editing, Supervision, Resources, Methodology. **Joanna Raven:** Writing – review & editing, Supervision, Methodology, Investigation, Formal analysis, Data curation, Conceptualization. **Rosalind McCollum:** Writing – review & editing, Writing – original draft, Supervision, Methodology, Investigation, Formal analysis, Data curation, Conceptualization. **Sally Theobald:** Writing – review & editing, Supervision, Resources, Formal analysis. **Wede Seekey:** Writing – review & editing, Writing – original draft, Methodology, Investigation, Formal analysis, Data curation, Conceptualization. **Jerry Kollie:** Writing – review & editing, Methodology, Investigation, Formal analysis, Data curation. **Hannah Berrian:** Writing – review & editing, Methodology, Investigation, Formal analysis, Data curation.

## Ethics

The study received ethical approvals from the Liverpool School of Tropical Medicine Research Ethics Committee, United Kingdom (protocol ID 20-040) and UL-PIRE’s Institutional Review Board, Liberia (protocol ID 20-09-233) in March and April 2020, respectively. All participants were provided with adequate information about the study and interview, and provided written consent. Photovoice participants received training about the ethics surrounding taking photographs, and were advised of the need to seek consent before taking someone’s photo. Where a person was recognizable in the photograph researchers ensured that informed consent for the use of their photo was taken. As part of the consent process, photovoice participants were given the option to have their name included alongside their photos, and so you will see the name of the photographer alongside some of the photos included within the paper where this consent was given.

## Funding statement

This work was supported and funded by the National Institute for Health Research (10.13039/100006662NIHR), [REDRESS: Reducing the Burden of Severe Stigmatising Skin Diseases through equitable person-centred approaches to health systems strengthening (Project reference NIHR200129)/Research and Innovation for Global Health Transformation]. The views expressed are those of the author(s) and not necessarily those of the NIHR or the Department of Health and Social Care.

## Declaration of Generative AI and AI-assisted technologies in the writing process

During the preparation of this work the authors did not use AI and AI-assisted technologies in the writing process.

## Declaration of Competing Interest

The authors confirm they have no conflict of interest.

## Data Availability

Data is presented within the manuscript. Any additional data required can be made available upon request.
